# Evaluation of a Developed MRI-Guided Focused Ultrasound System in 7 T Small Animal MRI and Proof-of-Concept in a Prostate Cancer Xenograft Model to Improve Radiation Therapy

**DOI:** 10.3390/cells12030481

**Published:** 2023-02-02

**Authors:** Xinrui Zhang, Sebastian Greiser, Upasana Roy, Franziska Lange, Robbert van Gorkum, Marc Fournelle, Daniel Speicher, Steffen Tretbar, Andreas Melzer, Lisa Landgraf

**Affiliations:** 1 Innovation Center Computer Assisted Surgery, 04103 Leipzig, Germany; 2 Fraunhofer Institute for Cell Therapy and Immunology and Fraunhofer Cluster of Excellence for Immune-Mediated Disease, 04103 Leipzig, Germany; 3 Institute for Biomedical Engineering, University and ETH Zurich, 8092 Zurich, Switzerland; 4 Fraunhofer Institute for Biomedical Engineering, 66386 St. Ingbert, Germany; 5 Institute of Medical Science and Technology, University of Dundee, Dundee DD1 4HN, UK

**Keywords:** magnetic resonance guided focused ultrasound, MRgFUS, prostate cancer cells, cancer treatment, hyperthermia, radiation therapy, radiosensitization

## Abstract

Focused ultrasound (FUS) can be used to physiologically change or destroy tissue in a non-invasive way. A few commercial systems have clinical approval for the thermal ablation of solid tumors for the treatment of neurological diseases and palliative pain management of bone metastases. However, the thermal effects of FUS are known to lead to various biological effects, such as inhibition of repair of DNA damage, reduction in tumor hypoxia, and induction of apoptosis. Here, we studied radiosensitization as a combination therapy of FUS and RT in a xenograft mouse model using newly developed MRI-compatible FUS equipment. Xenograft tumor-bearing mice were produced by subcutaneous injection of the human prostate cancer cell line PC-3. Animals were treated with FUS in 7 T MRI at 4.8 W/cm^2^ to reach ~45 °C and held for 30 min. The temperature was controlled via fiber optics and proton resonance frequency shift (PRF) MR thermometry in parallel. In the combination group, animals were treated with FUS followed by X-ray at a single dose of 10 Gy. The effects of FUS and RT were assessed via hematoxylin-eosin (H&E) staining. Tumor proliferation was detected by the immunohistochemistry of Ki67 and apoptosis was measured by a TUNEL assay. At 40 days follow-up, the impact of RT on cancer cells was significantly improved by FUS as demonstrated by a reduction in cell nucleoli from 189 to 237 compared to RT alone. Inhibition of tumor growth by 4.6 times was observed in vivo in the FUS + RT group (85.3%) in contrast to the tumor volume of 393% in the untreated control. Our results demonstrated the feasibility of combined MRI-guided FUS and RT for the treatment of prostate cancer in a xenograft mouse model and may provide a chance for less invasive cancer therapy through radiosensitization.

## 1. Introduction

Image-guided procedures have been gaining more and more clinical acceptance, especially in the field of cancer diagnosis and treatment. Imaging via ultrasound (US), X-ray computed tomography (CT), or magnetic resonance imaging (MRI) allows for minimal- and non-invasive procedures such as biopsies, thermal ablation, embolization [1], and focused ultrasound. Imaging guidance leads to smaller incisions or incisionless surgery and thus reduces the side effects of surgery and shortens the time of hospitalization.

MRI-guided focused ultrasound (FUS) or high-intensity focused ultrasound (MRgFUS/MRgHIFU) describes the technique of using ultrasound beams to heat a target tissue inside the body in a non-invasive way [2]. In contrast to diagnostic ultrasound, a special form or, e.g., an array of multiple transducers, bundles ultrasound waves into a focus point or focus volume for targeted therapy. Ablation of tissue at temperatures above 55 °C using HIFU is already approved by Food and Drug Administration (FDA) for the ablation of benign tumors of the uterus, for ablation of prostate tissue, in the palliative treatment of bone metastasis, and the therapy of essential tremors [3]. During these procedures, the treatment planning and control during therapy can be performed either by US-guidance (USgHIFU) or by MRI-guidance (MRgHIFU). In addition to these clinically approved applications, FUS preclinical studies for histotripsy, blood-brain-barrier opening, support of drug/gene delivery into a targeted region, and generation of hyperthermia have been reported [4]. 

Hyperthermia can be used in cancer treatments and is defined as the heating of tissue to a temperature range of 40–45 °C which is maintained at the treated site for a period of time [5]. Currently, radiofrequency or microwave-induced hyperthermia is in clinical use as an adjuvant treatment for cancer therapy with minimal adverse effects and adjacent tissue damage [6]. However, electromagnetic waves-based hyperthermia techniques cannot be focused as precisely as ultrasound waves, and the radiofrequency or microwave-hyperthermia systems are limited in penetration depth. 

FUS overcomes these limitations as it is based on mechanical pressure waves, such that a focal spot can be generated with an adequate penetration depth ranging from 1–20 cm. Hence, FUS-induced hyperthermia is becoming an attractive option. While FUS ablation has been proven for several clinical treatments, FUS-induced hyperthermia has not yet been approved. As the number of clinical applications of thermal therapies grows, it is becoming more and more relevant that FUS has demonstrated great potential in thermal therapies as part of multi-modality cancer treatment strategies [7]. 

Due to the availability of extensively developed tumor models, pre-clinical investigations in experimental small animals are widely employed to elucidate the efficacy and mechanisms of novel therapies [8]. Platforms for the reliable delivery of prescribed experimental thermal therapy protocols (e.g., desired time-temperature profiles) to small animal targets are crucial tools for facilitating these pre-clinical investigations. 

Prostate cancer is the second-most common cancer in men worldwide [9]. As one of the most commonly used and highly cost-effective treatment modalities, radiation therapy (RT) is typically applied as a single modality or as part of adjuvant therapy for the treatment of prostate cancer [10]. However, there is still a need for optimized treatment regimens using lower radiation doses while bringing equivalent outcomes or even higher survival rates. Normally, patients receive a total radiation dose of 70 Gy (2 Gy per day) in clinical practice, however, RT treatment is associated with urinary and bowel side effects. Thus, various targets for radiosensitization in prostate cancer were investigated to reduce the radiation dose and increase treatment outcomes [11]. 

The mechanisms of radiosensitization in cancer cells include the inhibition of repair of DNA damage, cell cycle arrest [12], change of prostate cancer cell gene expression [13], and reduction in tumor hypoxia [14]. In numerous studies, hyperthermia demonstrated sensitization effects to tumor cells and thus supports chemo- and radiotherapy effectively [15]. Here, the MRgFUS treatment is focused on the moderate heating of tumor tissue and enables treatment temperatures at lower levels around 40–47 °C. In this scenario, the combination of MRgFUS-induced hyperthermia and RT could represent a pivotal breakthrough to improve patient outcomes. In our previous in vitro study, a combination of FUS-induced hyperthermia and RT with a short time interval of 1 h showed an additive effect compared to a single treatment in prostate cancer cells [16]. For the realization of in vivo treatment and further translation, an MRI-conditional small animal FUS platform was designed and manufactured. 

In this work, a new multimodal small animal phased array transducer was developed and tested in a pre-clinical MRI scanner. MRI safety and image compatibility are important issues when using FUS Systems in the environment of MRI. MRI testing of medical devices is required for device approval in the U.S. and Europe. Moreover, individual safety concerns such as the safe functioning of the device and the MRI system are necessary for medical devices within an MRI environment. Standardized tests increase the safety of the patient and support both the MRI user and the device manufacturers. We measured the MRI compatibility, including field mapping of the static magnetic field, and checked the image quality in terms of signal-to-noise and image homogeneity. Furthermore, FUS protocols for radiosensitization were designed and proton resonance frequency (PRF) shift MR temperature mapping was performed offline in our own adopted software tool for analyzing the PRF and phase measurements.

## 2. Materials and Methods

### 2.1. FUS Electronics and Probe

**Figure 1:**
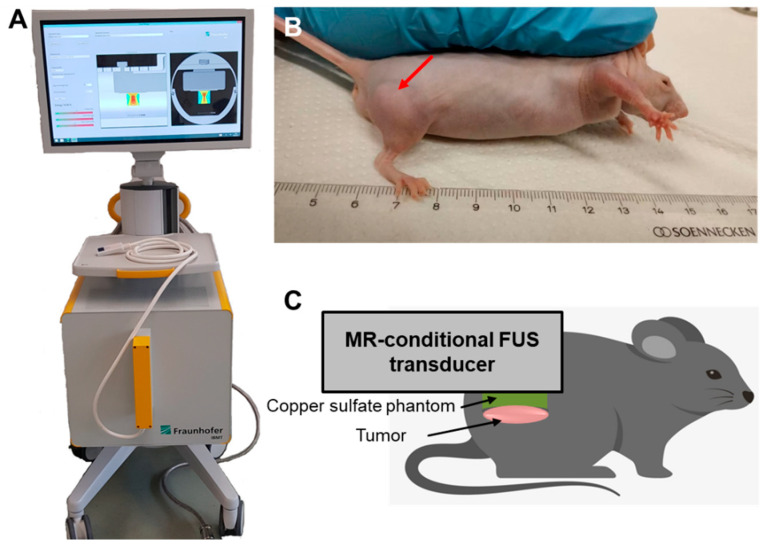
(**A**) FUS system and (**B**) the xenograft mouse model established by the subcutaneous injection of human prostate cancer PC-3 cells. Red arrow point to tumor. (**C**) Schematic of the experimental setup. The mouse was anesthetized with isoflurane and placed on a warming pad to keep the body temperature constant. The MRI-conditional FUS transducer was positioned on top of the subcutaneous tumor and the phantom pad and ultrasound gel were used for coupling.

Based on the available space in pre-clinical MRI systems and technical parameters needed for a FUS treatment, a novel MR-conditional preclinical FUS phased array transducer consisting of an 11 × 11 element matrix array probe with an aperture size of 10 × 10 mm^2^ and a frequency of 960 kHz was developed by Fraunhofer IBMT (Sulzbach & St. Ingbert, Germany) (Figure 1A). The system is described in detail in “A novel matrix-array-based MR-conditional ultrasound system for local hyperthermia of small animals” [17]. The matrix has a pitch of 909 µm in both directions with inter-element gaps of 100 µm realized by dicing. The piezoelectric array was mounted directly on a printed circuit board with a thin conductive backing layer to reduce the height of the acoustic block (in view of the severe size constraints resulting from the need for integration for the bore of the small animal MRI). In addition, the probe had an inner shielding with copper to improve the electromagnetic compatibility behavior. The acoustic block was integrated into a 3D-printed housing with an overall height of 11 mm. 

The transducer is driven by a modified version of the multichannel ultrasound research system DiPhAS [18]. The system has 128 transmit and receive channels, whereof only the transmit paths are used in the present work. Transmit signals can be parameterized with a transmit clock of 120 MHz within the limits of the tri-state pulser capacities (maximum voltage of +/−75 V). The electronics were modified to be able to generate higher duty cycles when compared with the basic system version designed for imaging tasks. In the setting used in the present work, bursts with a length of up to 50 µs at a pulse repetition frequency of 1 kHz could be generated. 

The focusing properties of the system could be adjusted via the user interface (UI) of the software which allows for defining the coordinates of the focus spot in (x/y/z). In addition, the UI allows for the setting of the transmit voltage of the system, the signal length, the pulse repetition frequency, and the sonication time. The acoustic output of the system depends on the chosen focus position. For instance, when focusing on (x/y/z) = (0/0/5) {mm}, the lateral extent of the pressure focus (−6 dB of the full-width half maximum FWHM) is 1.5 mm and the axial FWHM is 7.3 mm. When the transducer is driven with the maximum duty cycle of 1 kHz, the maximum burst length of 50 µs, and the maximum voltage of +/−75 V, the ISPTA was 4.8 W/cm^2^ and 3.8 W/cm^2^ when focusing on (x/y/z) = (0/0/5) {mm} and (0/0/10) {mm}, respectively. 

### 2.2. MRI-Compatibility of the FUS Transducer in 7 T MRI

**Figure 2:**
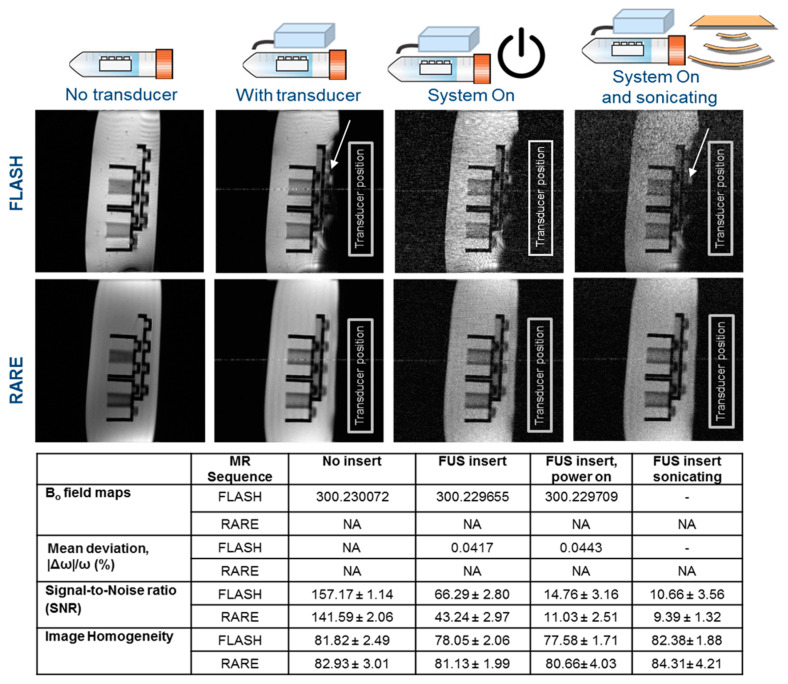
Image quality and results of MRI measurements of the phantom (Part No.: T10681, Bruker) consisting of Agar-Agar 10 g/L, CuSO_4_, 2H_2_O salt 1 g/L, and Lego bricks in 7 T preclinical MRI with and without FUS transducer. The arrows point to artifacts.

To test the MRI compatibility of the FUS transducer, the DiPhAS system was placed next to the MRI. The transducer adapter had a cable length of 1.5 m and the transducer was fixed with tape on a rat body animal bed (Part No.: T11611, Bruker, Ettlingen, Germany) of a 7 T preclinical MRI (PharmaScan 7 T, Bruker) scanner. MRI protocols were used according to the existing literature where measurements were carried out to test the MRI compatibility of external hardware [19]. A standard MRI phantom (Part No.: T10681, Bruker) consisting of Agar-Agar 10 g/L, CuSO_4_, 2H_2_O salt 1 g/L, and two Lego bricks were used for imaging. The FUS transducer and the phantom were placed on the rat bed and a 60-mm-diameter transmit and receive coil (rat whole body resonator, Part No.: T11527V3, Bruker) was used. To test for imaging compatibility, MR images were obtained with the fast low-angle shot (FLASH) gradient echo sequence (Parameters: echo time (TE) = 15 ms; repetition time (TR) = 400 ms; slice thickness = 3 mm; field of view FOV = 4 × 4 cm^2^; matrix = 256 × 256 pixels; flip angle (FA) = 30°; and no. of averages = 1). With all these parameters, the total scan time was three minutes. Additionally, a rapid imaging of refocused echo (RARE, comparable to turbo/fast spin-echo) sequence was run with the same geometry, except TE = 20 ms and TR = 800 ms. MR imaging was repeated with the same geometry under four different conditions (e.g., Figure 2): (a) only the phantom and no transducer on the rat bed, (b) the FUS transducer and the phantom on the rat bed but the DiPhAS was not powered, (c) the FUS transducer and the phantom on the rat bed and the DiPhAS was powered, and (d) FUS transducer sonicating.

### 2.3. Acquiring B_0_ Field Maps

B_0_ field maps represent the frequency shift from the Larmor frequency and are scaled from −300 to 300 Hz, roughly spanning ±1 ppm relative to the Larmor frequency at 7 T (~300 MHz) and giving feedback about the homogeneity of the magnetic field. To calculate the B_0_ field map, the raw image data with gradient echo sequence at two different echo times are required. We assume that image m_1_(x, y) is acquired at time TE_1_ and image m_2_(x, y) is acquired at TE_2_. 

Then, the phase (φ) difference in the two images will be,

φ_1_ − φ_2_ = angle (m_1_* · m_2_) (1)

where the complex conjugate of m_1_ is denoted as m_1_*. In a gradient echo sequence, if the spins have an initial phase (φ_0_), then after echo time TE_1_,

Phase, φ_1_ = φ_0_ + ω · TE_1_ (2)

After echo time TE_2_,

Phase, φ_2_ = φ_0_ + ω · TE_2_ (3)

Overall phase difference,

φ_1_ − φ_2_ = ω · (TE_1_ − TE_2_) = γ · B_0_ · (TE_1_ − TE_2_) (4)

A comparison of Equations (1) and (4) gives,

ω = (φ_1_ − φ_2_)**/**(TE_1_ − TE_2_) = angle (m_1_* · m_2_)**/**(TE_1_ − TE_2_) (5)

where ω is the precession frequency of spins in the external magnetic field (MHz), B_0_ is the strength of the externally applied magnetic field, and γ is the gyromagnetic ratio of the spin. The B_0_ field maps provide the fluctuation of the magnetic field with and without the transducer. To calculate this, a FLASH sequence was performed with the Agar-Lego phantom in Bruker 7 T MRI with two echo times: 10 ms and 20 ms. Other parameters: TR = 400 ms; slice thickness = 2 mm; FOV = 4 × 4 cm^2^; matrix = 256 × 256 pixels; FA = 20°; no. of averages = 4; and inter-slice distance = 0.5 mm. With all these parameters, the total scan time was 5 min. Field maps were generated using custom MATLAB (MathWorks, Natick, MS, USA) scripts. In order to assess the field homogeneity over the entire phantom volume, regions outside of the phantom with low SNR were cropped.

### 2.4. Image Analysis

The signal-to-noise ratio (SNR) was calculated using the mean signal in the region of interest (ROI), S divided by the standard deviation of noise in a background ROI, and σ (avoiding any possible phase-encoding artifacts) with a correction factor to account for the Rician noise distribution of magnitude images in the MRI:

SNR = 0.655 · (S/σ)(6)

Image homogeneity was calculated as the normalized difference between the maximum signal, S_max_ and minimum signal, S_min_ in each ROI:

[1 − (S_max_ − S_min_)/(S_max_ + S_min_)] · 100% (7)

SNR and image homogeneity were measured in all 13 slices spanning the phantom and the mean and standard deviation were calculated for each slice using repeated image acquisitions (n = 3) of the Bruker 7 T MRI. 

### 2.5. Proton Resonance Frequency Shift MR Thermometry

The non-invasive measurement and mapping of the spatiotemporal distribution of tissue temperature are essential for the development and application of MRgFUS. MR thermometry is based on several temperature-sensitive MRI parameters such as the proton resonance frequency (PRF), the diffusion coefficient (D), T1 and T2 relaxation times, magnetization transfer, and the proton density, as well as temperature-sensitive contrast agents. In this work, temperature maps were acquired based on the proton resonance frequency (PRF) and phase measurements, hereafter referred to as PRF thermometry. The resonance frequency of water protons is affected by temperature and can be expressed as:

f_water_ = γ(1 + σ_0_ + σ_T_)B_0_ (8)

σ_T_ = α · ΔT (9)

where σ_T_ and σ_0_ represent the local magnetic field changes caused by temperature and non-temperature factors, B_0_ is the static magnetic field strength, γ is the gyromagnetic ratio, ∆T is the temperature change (°C), and α is the temperature-dependent coefficient (ppm/°C). The resonance frequency change resulting from temperature can be written as:

Δf = γ · α · ΔT · B_0_ (10)

Clearly, at a higher field strength B_0_, the same temperature change ∆T would lead to a greater ∆f, indicating that higher temperature sensitivity (or TNR) can be achieved. For a given echo time (TE, unit: ms), the accumulated phase variation (or phase difference) ∆ϕ(x, y) (unit: °) at position (x, y) during TE is:

Δϕ(x, y) = Δf · TE = γ · α · ΔT · B_0_ · TE (11)

Or in another form

ΔT = Δϕ(x, y)**/**γ · α · B_0_ · TE = Δϕ(x, y) · α · f_0_ · TE (12)

where f_0_ represents the resonance frequency (MHz). Equation (12) suggests that the temperature change could be estimated from the phase difference image ∆ϕ(x, y) when α is known. The value of α is considered to be −0.01 ppm/°C. 

### 2.6. PRF Thermometry in Tissue-Mimicking Phantoms (TMP)

A tissue-mimicking (TM) material for ultrasound, using evaporated milk as the primary absorption component along with Agar, was prepared. This phantom has a very low backscatter but still exhibits the 1540 m s^−1^ propagation speed and proportionality of the attenuation coefficient and frequency over the diagnostic frequency range. The detailed steps for the phantom preparation were adapted from Madsen et al. 1998 [20]. 

To begin, single-step FUS sonication was performed followed by PRF thermometry. The TMP was sonicated for 2 min with 2.4 W/cm^2^ at a focus spot (0/0/5 mm) along the sonication direction. MRI experiments were performed with the 7 T Bruker scanner before and after this sonication step. T2-weighted reference images were acquired using a multi-slice spin echo scan with five axial, sagittal, and coronal slices centered on the light spot. The scan parameters were as follows: Slice thickness = 3 mm, inter-slice distance = 0.5 mm, FOV = 5 × 5 cm^2^; TE = 20 ms; TR = 500 ms; matrix = 256 × 256 pixels; and acquisition time = 20 s. The T1 and T2 values of the phantom were determined with RARE relaxometry acquisitions with the following parameters: TE = 7–105 ms; RARE factor 2; and TR = 80–5000 ms. T2 values were determined with a multi-gradient echo sequence with the following parameters: TE_min_ = 2.9 ms; ΔTE = 7.0 ms; TE_max_ = 60.4 ms; TR = 1500 ms; and FA = 30°. For PRF thermometry, a FLASH sequence was used with TE approximately equal to T2 (typically between 7 and 10 ms), a matrix of 128 × 128 pixels, and a flip angle equal to the Ernst angle (typically between 12° and 14°). Other scan parameters were similar to the anatomical images. 

The experimental protocol for a real in vivo experiment requires a slow increase in the temperature at the targeted FUS heating site. This step has to be monitored with PRF thermometry. This implies that MR images have to be recorded after each sonication step. During the heating period, each FUS sonication section was manually started with 4.8 W/cm^2^ at a focus spot (0/0/5 mm) for 55 s followed by MRI. During the cooling period, the interval between two consecutive MR image acquisitions was kept the same as the heating period but the sonication was omitted in order to reduce the temperature of the FUS target. 

### 2.7. PRF Thermometry in Mice (Ex Vivo)

The first measurement of FUS heating efficiency was performed by targeting the hindlimb of a freshly euthanized nude mouse. Warm water (36 °C) was pumped through a rubber warming pad (Part No.: T10965, Bruker) underneath the mouse to avoid a strong temperature decrease by the cooling system of the MRI gradient coil. A 0.6 mm thick agar pad was placed between the FUS transducer and the target tissue. This way, a signal loss of MRI at the transducer site and ringing artifact at the surrounding tissue could be avoided. The time-series FUS sonication sections were manually started for 55 s with an intensity of around 4 W/cm^2^ at the focus spot (0/0/7.5 mm) and followed by MR imaging. The intensity of the FUS sonication was measured beforehand with 4.8 W/cm^2^ for (0/0/5 mm) and 3.8 W/cm^2^ for (0/0/10 mm). The FLASH parameters were: TE = 4.5 ms; TR = 275 ms; FOV = 8 × 5 cm^2^, 15 slices with thickness of 1.5 mm; matrix = 256 × 256 pixels; and acquisition time = 52 s. Real-time temperature monitoring at the FUS target was performed with a fiber optic probe (Luxtron). PRF thermometry was visualized offline by reconstructing k-space data in MATLAB [21].

### 2.8. Cell Culture

Human prostate cancer cell line PC-3 was purchased from the European Collection of Authenticated Cell Cultures (ECACC, Salisbury, UK). Cells were cultured in Ham’s F-12K (Kaighn’s) Medium (F12K) supplemented with 10% fetal bovine serum (FBS), 100 U/mL penicillin, and 100 µg/mL streptomycin at 37 °C in a humidified atmosphere under 5% CO_2_ in an incubator. Cells were washed with phosphate-buffered saline (PBS, Biozym Scientific GmbH, Hessisch Oldendorf, Germany) and passaged with trypsin/EDTA (Biozym Scientific GmbH, Hessisch Oldendorf, Germany). Cells were routinely tested for mycoplasma.

### 2.9. Preparation of Mouse Xenografts

The experiments were performed using 7–15 weeks-old male NMRI Foxn1nu/nu mice (Janvier Labs, Le Genest-Saint-Isle, France). Mice were housed in the animal facility of Fraunhofer Institute for Cell Therapy and Immunology (Fraunhofer IZI) according to European (Council Directive 86/609/EEC) and German guidelines (Tierschutzgesetz) for the welfare of experimental animals. All experiments had been approved by the local authorities (Landesdirektion Sachsen TV29/18).

The xenograft prostate cancer model was generated by injection of PC-3 cells as described by Liebscher et al. [22]. Briefly, 0.5 million PC-3 cells suspended in 100 µL matrigel (Matrigel matrix growth factor reduced, Corning Inc., New York, NY, USA) were transplanted subcutaneously into the right hind leg of mice (Figure 1B).

### 2.10. In Vivo MRI

For in vivo MRI measurements, the mice were anesthetized with 2% isoflurane (AbbVie Inc., North Chicago, IL, USA) in the air (0.5 L/min) and 20% oxygen; the positioning of the mice during treatment is shown in Figure 1C. During MRI scanning, the level of anesthetic was maintained between 2% and 2.5% to keep the breathing of the animal at a constant rate of ~60 breaths per minute. The respiration rate was instantly monitored using a respiration sensor placed on the animal’s abdomen and connected to a respiration unit, connected to a computer with Bio-SAM respiration monitoring software (SA Instruments, Stony Brook, NY, USA). To prevent cooling of the animal during the intervention, the mouse’s body temperature was kept at a constant physiological temperature by pumping 36 °C warm water through a warming pad (T10965, Bruker) underneath the mouse. 

### 2.11. MRgFUS Treatment In Vivo

The major goal with the new FUS setup is to perform the thermal treatment at a temperature of 45 °C for 30 min. Therefore, the temperature of the target region needs to be increased and held at 45 °C. With the 2 MHz transducers, the sonication power was kept at maximum (4.8 W/cm^2^) for the first 2 min so that the temperature of the targeted tumor was raised from 37 to 45 °C. Afterward, the power was reduced and varied between 10–15% so that the temperature was stable at 45 °C for 30 min. The temperature profile was obtained by PRF thermometry and a fiber optic probe attached to the skin of the tumor measured the real-time temperature as a reference.

### 2.12. Radiation Therapy

For single radiation and in the combination group, the mice were anesthetized with isoflurane and then transferred into the small animal radiation research platform (SARRP, XStrahl Ltd., Camberley, UK). The single dose X-ray irradiation of subcutaneous tumors was performed at 10 Gy (220 kV, dose rate: 2.53 Gy/min) and the animals were returned to the cages afterward and observed until complete consciousness was regained. In the combination group, irradiation at 10 Gy was performed within one hour after FUS treatment.

### 2.13. Study Design

In the orientation study, 16 mice were divided into control, FUS, RT, and FUS + RT groups (n = 4 each). Single FUS and RT treatment was performed 4 weeks after tumor transplantation according to the protocol described above. In the FUS + RT group, the animals were treated first by FUS-induced hyperthermia at 45 °C for 30 min, and single-dose irradiation at 10 Gy was delivered afterward within 1 h. Animals were weighed using an electronic balance and the tumor sizes were measured with a sliding caliper three times per week. Animals were sacrificed if they appeared to suffer when the tumor size reached a diameter of 15 mm or after reaching a follow-up time of 40 days after treatment. The tumor volume was calculated as *V* = *π*/6 · *a* · *b*^2^, where *a* is the longest and *b* is the perpendicular shorter tumor diameter.

### 2.14. Hematoxylin and Eosin (H&E) Staining

For a morphological overview of cell nuclei and cell membranes, H&E staining was performed. Subcutaneous tumors and organs (lung, liver, kidney, and spleen) were collected from all animals and fixed in 4% formaldehyde (Carl Roth GmbH, Karlsruhe, Germany) for 24 h and infiltrated in 30% sucrose solution in PBS for 2–3 days. Tissues were snap-frozen in isopentane (Carl Roth) at −60 °C on dry ice. Cryosections with a thickness of 8 µm were obtained using a Leica CM3050S Cryostat (Leica Microsystems, Wetzlar, Germany). The H&E staining was performed for general histology in the automatic staining system (Leica ST5020, Leica Microsystems). The images were acquired using a Keyence microscope (BZ-9000, KEYENCE GmbH, Neu-Isenburg, Germany) at 40-fold magnification, and tumor nucleoli in each section were counted using the cell count function in ImageJ [23]. 

### 2.15. Immunohistochemistry Ki67

For the quantification of proliferated tumor cells, the immunohistochemistry of Ki67 was performed. Briefly, the cryosections of tumors were thawed at room temperature, boiled in sodium citrate at 80 °C for 20 min, and then blocked in a 10 mM sodium azide buffer supplemented with 0.1% H_2_O_2_ in tris-HCl for 1 h. Afterward, samples were incubated in primary Ki67 recombinant rabbit monoclonal antibody (SP6, Thermo Fisher Scientific, Dreieich, Germany) at a dilution of 1:1000 at 4 °C overnight. Samples were washed three times in tris-washing buffer and incubated with goat anti-rabbit IgG antibody (H + L), biotinylated secondary antibody (Vector Laboratories, Inc., Burlingame, CA, USA) at 37 °C for 2 h. The staining procedure was performed according to the manufacturer’s instructions by applying the VECTASTAIN ABC-HRP kit (Cat#PK-4000, Vector Laboratories Inc, Burlingame, CA, USA). Visualization was performed with an ImmPACT DAB peroxidase substrate kit (Vector Laboratories, Inc., Burlingame, CA, USA) and counterstained with hematoxylin. An automated slide scanner (ZEISS Axio Scan.Z1, Carl Zeiss Microscopy GmbH, Jena, Germany) with a 10-fold objective (Plan-Apochromat 10×/0.45 NA) was used for imaging. For quantification, the immunostained cells and the total cell number in three random fields of each section were counted in QuPath software [24]. The percentage of positive cells was used as an indicator of tumor proliferation.

### 2.16. Apoptosis Assay

A terminal deoxynucleotidyl transferase dUTP nick end labeling (TUNEL) assay was performed for the detection of tumor apoptosis using the in situ cell death detection kit (Cat#11684795910, Sigma-Aldrich GmbH, Munich, Germany) according to the manufacturer’s protocol. Apoptotic DNA fragmentation was labeled by 3′-hydroxyl termini and tumor nucleoli counterstaining was performed with DAPI (Thermo Fisher Scientific, Dreieich, Germany). Images were captured under a fluorescence microscope (ZEISS Axio Observer.Z1) using a 40-fold objective. The fluorescent surface area of each section was quantified in ImageJ [23].

### 2.17. Statistical Analysis

Each group contained four animals (N = 4) and 2–3 cryosections of each tumor were stained for quantification. All data of tumor nucleoli (H&E staining), tumor proliferation (immunohistochemistry staining of Ki67), and apoptosis (TUNEL assay) were expressed as the mean ± SEM (standard error of the mean). The significance of the difference between the two groups was assessed by a one-way ANOVA test and Tukey test for post-hoc analysis in SPSS statistic software version 24. A *p*-value ≤ 0.05 was considered statistically significant.

## 3. Results

### 3.1. FUS Transducer MRI-Compatible for In Vivo Treatment

MRI-compatibility measurements included field mapping of the static magnetic field (B_0_) and the image quality from acquisitions with rapid imaging of refocused echo (RARE) and fast low-angle shot gradient echo (FLASH). The comparison of the MR image quality in the 7 T preclinical MRI with and without the transducer is shown in Figure 2. Images with reduced SNR were observed in the presence of the transducer. 

B_0_ field maps revealed a small degradation in the mean homogeneity when the FUS transducer was installed. MR images of the Agar-Lego phantom showed a field drift of 0.0417% with the presence of the transducer. A field drift of 0.0443% was observed when the transducer was sonicating. Without the transducer, the SNR was 157.1 ± 1.1 when using the FLASH sequence. Measurements on identical geometry with the presence of the transducer resulted in images with an SNR of 66.3 ± 2.8. When the DiPhAS system power was turned on, the SNR was further reduced to 14.8 ± 3.2. At last, the sonication with the FUS transducer was started and the SNR was reduced to 10.7 ± 3.6. In addition, there was a signal loss in the transducer site. With the RARE sequence, no signal loss at the transducer site was observed. However, the SNR was reduced from 141.6 ± 2.1 to 9.4 ± 1.3 when the FUS transducer was sonicating. A zipper artifact with a thickness of 0.8 mm was also present in both FLASH and RARE sequences and passed through the center of the image along the phase-encode direction. 

**Figure 3:**
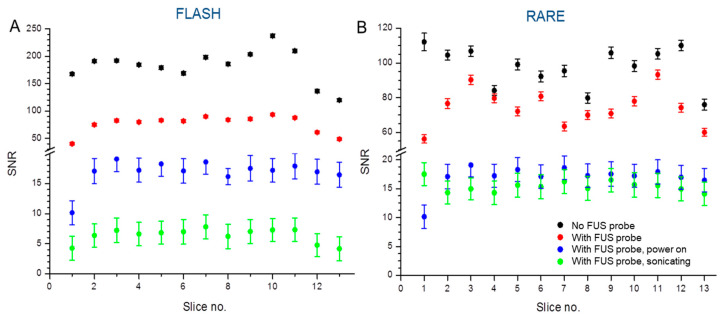
Measured mean signal-to-noise ratio profiles in 7 T of FLASH and RARE sequences of the phantom. (**A**) FLASH sequences show consistent values across all 13 slices. (**B**) RARE sequences show inconsistent image homogeneity for the slices located at the very beginning and very end of the phantom. (The position of the phantom and the transducer are shown in Figure 2.) The data between groups are significantly different *p* ≤ 0.05.

Mean SNR profiles averaged over the 15 repetitions of the FLASH and RARE sequences show consistent values across all 13 slices for four set-ups (Figure 3). Only the RARE sequence showed inconsistent image homogeneity for the slices located at the very beginning and very end of the phantom. 

### 3.2. PRF Thermometry with Tissue Mimicking Phantom Showed Temperature Increase after Single Sonication 

**Figure 4:**
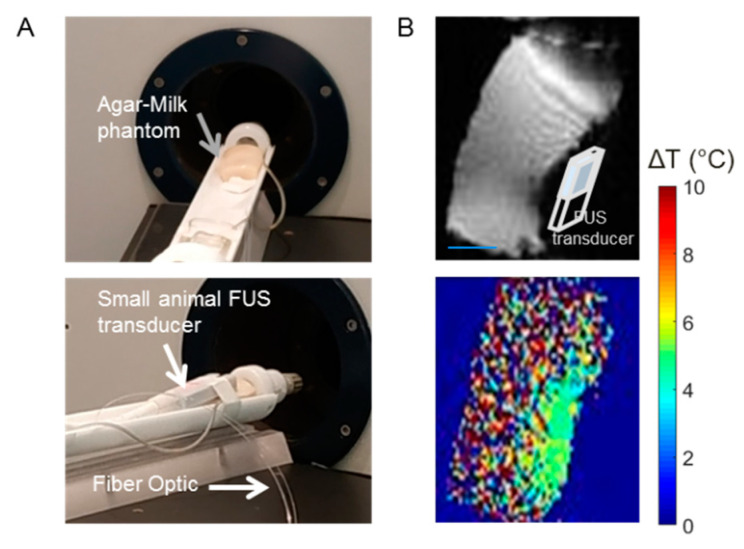
PRF thermometry on the tissue-mimicking phantom showed a temperature increase after a single sonication for 2 min. (**A**) Experimental set-up and the positioning of the phantom and FUS transducer. (**B**) The magnitude image of the phantom in sagittal orientation and the respective PRF thermometry color map.

The T_1_ and T_2_ relaxation times of the phantom were 283 ms and 75 ms, respectively. Therefore, the MRI parameters for the PRF thermometry provide proton density-weighted images. This means the images will have a high SNR but less contrast-to-noise within a small acquisition time. Figure 4A shows the position of the phantom and the transducer inside the rat bed of Bruker 7 T MRI. The magnitude images of the phantom in the sagittal direction are shown in Figure 4B. After a single sonication of 2 min with power 2.4 W/cm^2^ at a focus spot (0/0/5 mm), the temperature increase was calculated to be up to 7 °C. The temperature map from PRF thermometry was obtained and the heat signature was then plotted with a color map.

### 3.3. PRF Thermometry Measurements Conforms with Temperatures of Fiber Optic Measurements during Sonication in a TMP

**Figure 5:**
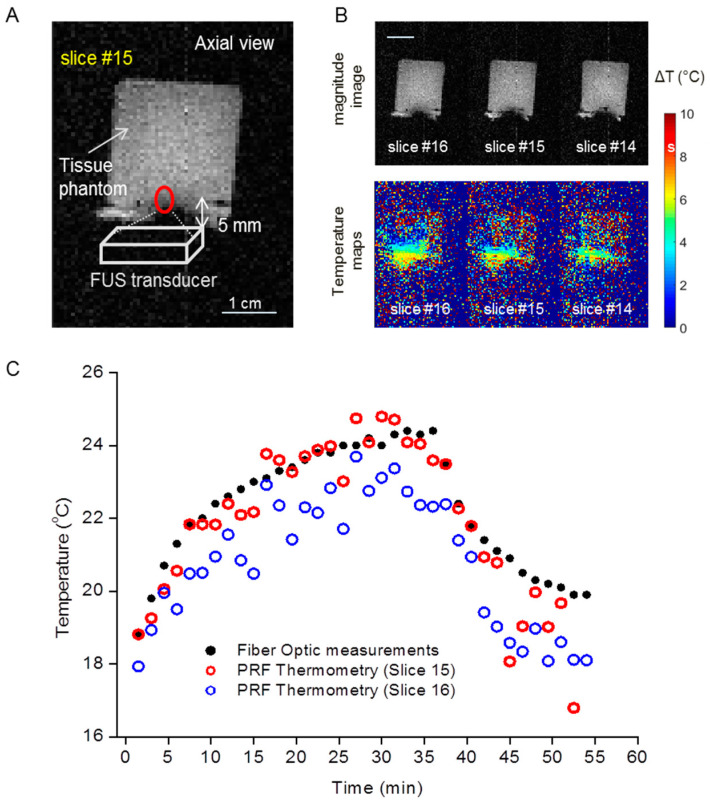
Temperature measured by fiber optic and PRF thermometry is consistent in the TMP. (**A**) Positioning of the FUS transducer and magnitude image of TMP. Red circle show the focus point of the transducer. (**B**) Temperature mapping of different slices after a series of sonications for 30 min. (**C**) The PRF thermometry measured temperatures are consistent with the temperatures measured by the fiber optic probe.

Additional phantom experiments were performed for time-series FUS sonication. Figure 5A shows the position of the transducer and the 15th slice of the phantom which contained the FUS focus. Since the image quality was reduced when the transducer was sonicating (Figure 2), MR images were obtained when FUS sonication was paused. The magnitude and temperature images from the phantom are shown in Figure 5B. The signal loss at the transducer site is presented in the magnitude image. The maximum temperature closest to the transducer was 7 °C after 30 min of sonication according to the sonication plan. Figure 5C shows the comparison of the temperatures measured with fiber optic probes and PRF thermometry. For the 15th, slice the maximum discrepancy is 0.8 °C between the fiber optic (24.3 °C) and the PRF thermometry (23.5 °C) during the heating step. As expected, the temperature at the surrounding slice had a smaller temperature increase compared to the slice containing the FUS focus. For example, in the 16th slice, the increase in temperature was ~1 °C less compared to the 15th slice.

### 3.4. MRI-Compatible FUS Transducer Allows for Tissue Heating of Ex Vivo Tumors

**Figure 6:**
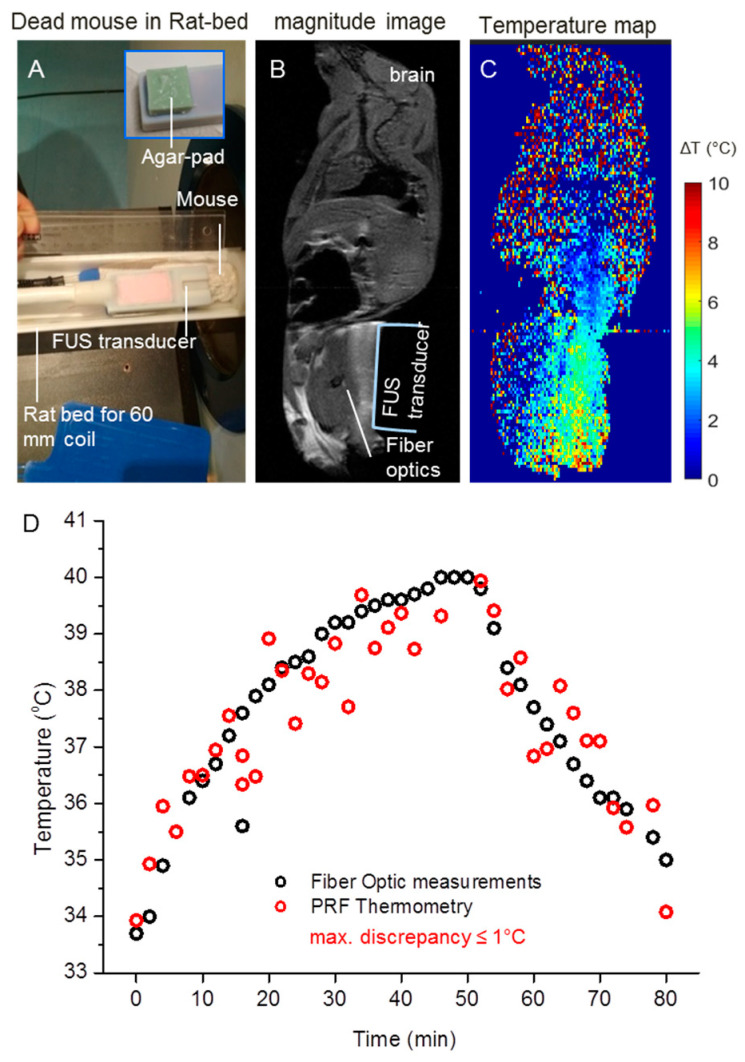
Temperature measurement in ex vivo mouse model. (**A**) Positioning of the mouse, FUS transducer, and agar pad in between. A rat bed and a 60 mm volume coil were used. (**B**) The magnitude image in sagittal orientation shows almost the whole mouse and the positions of the FUS transducer and the fiber optic probe. (**C**) The PRF thermometry color map demonstrates a temperature increase of 6 °C after 50 min of FUS sonication. (**D**) Temperatures from PRF thermometry and the fiber optic probe with a maximum discrepancy of 1 °C.

Figure 6A shows the position of the transducer and the mouse in the rat bed. A heating pad was placed below the euthanized mouse to keep the temperature of the mouse constant at 33 °C. Anatomical images of the mouse along the sagittal direction had an SNR of 21 (Figure 6B). The location of significant anatomical landmarks, i.e., brain, heart, spinal cord, liver, and kidneys were identified from different slices of the mouse body. The position of the transducer and the agar pad were also visualized. Due to the agar pad, no signal loss from the target tissue at the transducer site was observed in the magnitude image. Calculated temperature maps corresponding to a time-series FUS sonication of 50 min are shown in Figure 6C. Due to the agar pad, a slight heat dispersion was observed, but the target location of the FUS heating, i.e., the hind limb, was still heated. Figure 6D shows the comparison between the temperatures measured with the fiber optic probe and PRF thermometry. A maximum discrepancy of ≤1 °C was observed between the two measurements (38.5 °C in the fiber optic probe and 37.5 °C with PRF thermometry). 

### 3.5. MRI-Guided FUS Heating of Tumors up to 45 °C in a Xenografted Tumormouse Model

**Figure 7:**
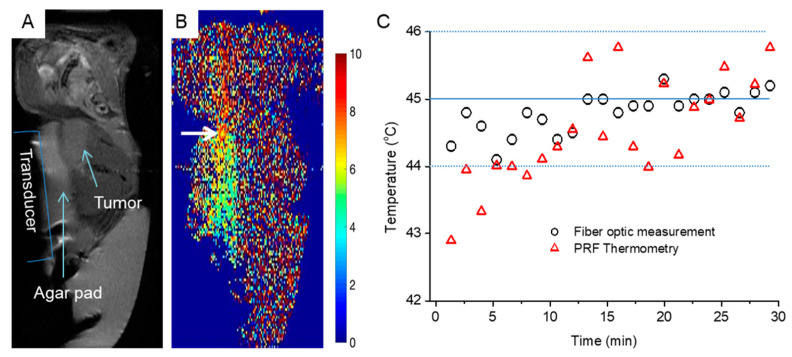
Experimental set-up and temperature mapping of MRgFUS treatment in a tumor-bearing mouse. (**A**) Positioning of the tumor-bearing mouse, agar pad, and FUS transducer. (**B**) PRF Temperature mapping. White arrow indicate tumor. (**C**) Diagram representing the PRF temperature mapping in comparison to the fiber optic probe during MRgFUS treatment for 30 min.

Figure 7A shows the position of the transducer, the agar pad, and the tumor in the magnitude image. The xenografted tumor has a diameter of 1.5 cm along the major axis. A heating pad was placed below the dead mouse to keep the temperature of the mouse fixed at 37 °C. The anatomical images of the mouse along the sagittal direction had an SNR of 13. Afterward, the sonication was started with the MRgFUS treatment protocol as described in 2.11 Figure 7B shows the temperature profile after the first 2 min of sonication with a fast increase from 37 °C to 45 °C. After this step, the power was reduced to keep the temperature at 45 °C for 30 min. Once again, the maximum temperature discrepancy between the fiber optic probe and PRF thermometry was measured to be 1.4 °C (45.2 °C in the fiber optic probe and 43.8 °C with PRF thermometry).

### 3.6. Combination Treatment of FUS and RT Leads to Decreased Tumor Growth

**Figure 8:**
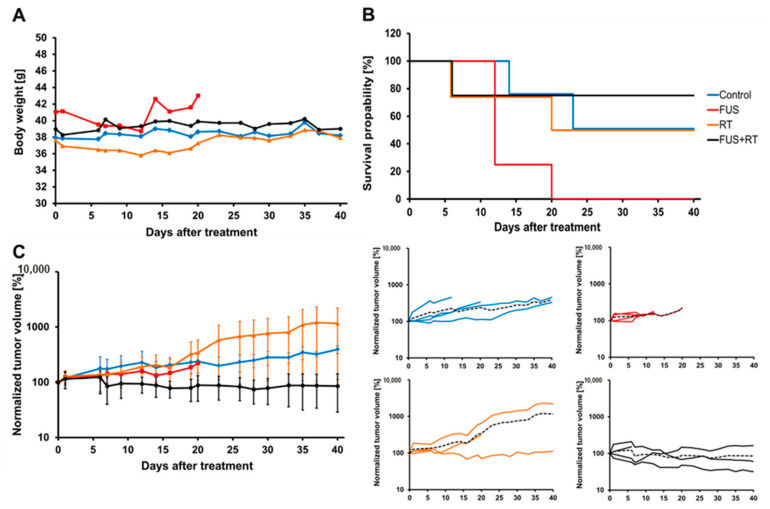
In vivo tumor growth of a prostate cancer (PC-3) xenograft was inhibited after a combination of FUS and RT treatment. The body weight of the animals was monitored three times per week. (**A**) During the follow-up period, mice treated with FUS and RT showed no significant body weight loss compared to the untreated control group (*p* > 0.05). (**B**) The combination of FUS and RT treatment enhanced the survival rate. (**C**) The tumor growth was suppressed by FUS + RT treatment, showing a decreased trend in tumor volume after treatment compared to RT alone.

The animals, which were treated with the in vivo FUS system (45 °C, 30 min), showed a similar body weight in comparison to untreated animals at day 20 (Figure 8A). Mice treated with single FUS (only FUS treatment) showed no obvious lesions in the important organs such as the lung, liver, kidney, and spleen. In contrast, a slightly decreased body weight was observed in the single RT group on day 13 but was enhanced to the normal level in the following days. Moreover, H&E staining demonstrated that the single FUS treatment did not lead to obvious histological changes in these organs compared to the untreated group. After FUS + RT treatment, the survival was higher compared to single treatments and the control group (Figure 8B). In addition, the tumor volumes in the FUS + RT group were significantly lower in comparison to the RT-only group (Figure 8C). Generally, the tumor growth showed a decreased trend after FUS + RT treatment and no regrowth of the tumor was observed in 40 days.

### 3.7. Histological Changes Prove Positive Effects of Combination Therapy of FUS and RT

**Figure 9:**
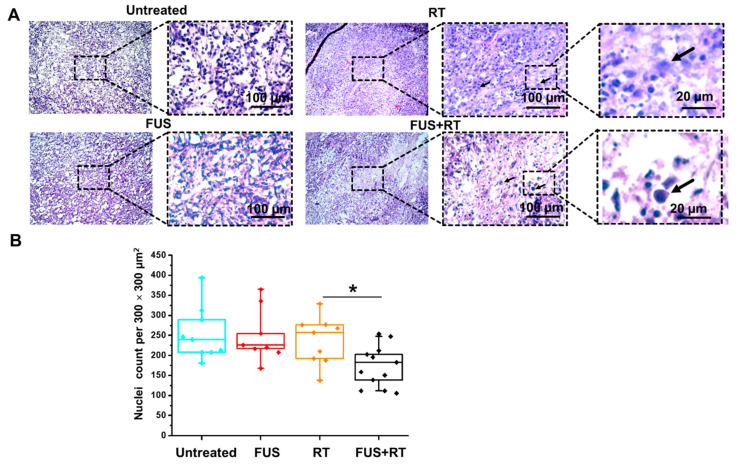
A combination of FUS and RT treatment leads to the membrane damage of tumor cells and a reduction in tumor nuclei number. (**A**) Hematoxylin-eosin staining showed the morphology changes of tumor cells after a single RT and a combination of FUS and RT treatment. Enlargement of the nuclei and distortion of the tumor cell membrane was observed in the single RT and FUS + RT group (black arrow). (**B**) The number of stained nuclei was measured in ImageJ using the cell counting function. The number of tumor nuclei was significantly reduced in the FUS + RT group compared to the single RT group (* *p* ≤ 0.05).

Generally, the H&E stained sections of tumor tissue from mice after RT and FUS + RT demonstrated histological alterations compared to the untreated control. Distinct damage of individual nuclei with distortion of the tumor cell membrane was observed in the single RT group (Figure 9A). Similar morphology changes in tumor cells were noticed in the FUS + RT group. The total number of tumor cell nuclei in the FUS + RT group (189 ± 14 nuclei) was significantly reduced compared to the RT-only group (237 ± 34 nuclei) (Figure 9B). Additionally, the overview of the section of the FUS + RT group revealed that the lesion in the tumor tissue is not uniform in comparison to the RT group.

### 3.8. Combination Treatment Suppresses Proliferation and Causes Apoptosis in PC-3 Cells

**Figure 10:**
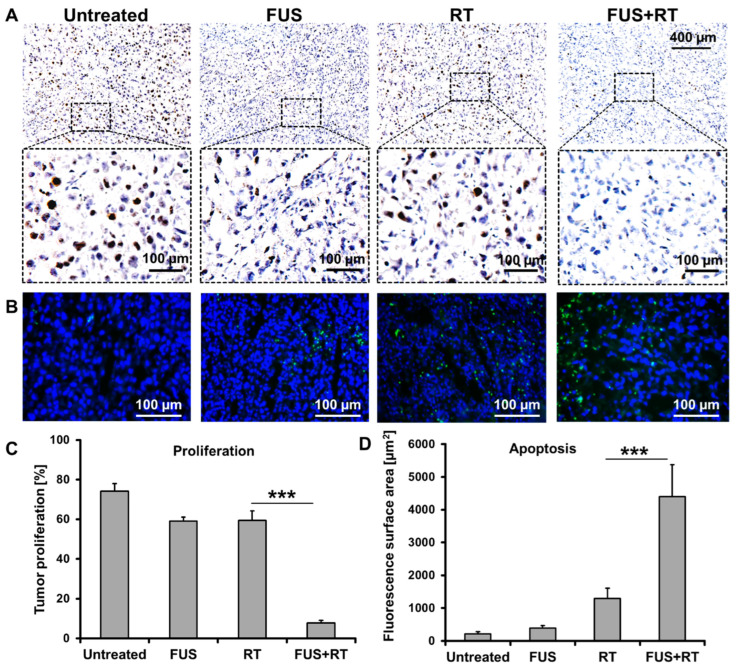
The prostate cancer xenograft tumor showed suppressed proliferation and enhanced apoptosis after FUS + RT compared to single RT. (**A**) Representative immunohistochemistry staining and (**C**) quantification of Ki67 demonstrated that the tumor proliferation was inhibited after a combination treatment of FUS and RT. (**B**) Fluorescence microscopy images of the TUNEL assay and (**D**) quantified fluorescence surface area show tumor apoptosis after treatment (green: apoptosis; blue: nucleus). N = 4 (*** *p* ≤ 0.001).

The expression of Ki67 was assessed as a marker of tumor proliferation in the xenograft prostate cancer model. We observed that the treatments with single FUS or RT led to a significant reduction of up to 40% in the percentage of Ki67-positive cells compared to the untreated control (74.1%; Figure 10A). As expected, the combination treatment of FUS + RT demonstrated additional benefits in comparison to single RT (59.4%), showing dramatically decreased proliferation to 7.9% (Figure 10C).

Samples were analyzed to detect the localized green fluorescence of apoptotic cells and blue fluorescence of cell nuclei using fluorescence microscopy (Figure 10B). The green fluorescence is almost not seen in the untreated group, and the green fluorescence-stained area is limited in the single FUS group. In contrast, the green fluorescence-stained area in the RT group is higher compared to that in the single FUS group. The largest green-stained area was observed in the FUS + RT group and is 3.6-fold higher compared to the single RT group (Figure 10D). In addition, the green fluorescence in the cryosections is not uniformly distributed, especially in the single FUS and FUS + RT groups.

## 4. Discussion

MRI-compatibility measurements included field mapping of the static magnetic field (B_0_) and acquisitions with multiple pulse sequences: RARE and FLASH. The SNR was influenced by the FUS transducer. Overall, there is a ~92% signal loss when the transducer is placed on the animal bed and inside the MRI. Similar findings were observed for the MRI compatibility tests with external hardware. For example, the installation of a PET insert at the 9.4 T Bruker pre-clinical scanner resulted in 14% SNR loss and 26% ghosting artifacts on the images [25]. Another study of MRI compatibility [19,26] reported 69% SNR loss when a new PET insert was installed. In all cases, the optimal settings were investigated for the best possible signal, and accordingly, the desired experiment was planned. Altogether, the developed small animal FUS transducer is MRI-conditional to allow safe performance of MRI-guided FUS treatment in a high-field 7 T magnet with a small bore of small animals such as mice. In regard to clinical translation, the use of the system will be possible in 3 and 1.5 T MRI scanners but should be then performed with bigger animals such as swine models or in human patients where such a high anatomical resolution for mice in 7 T is not necessary.

Due to its non-invasiveness, high-field PRF shift MR thermometry can offer attractive clinical benefits for the real-time monitoring of the temperature during an image-guided intervention [27]. We designed and tested a FUS set-up capable of inducing hyperthermia inside a 7 T small animal scanner and validated the MRI-derived temperature in tissue phantoms, ex vivo, and in an in vivo tumor-bearing mouse study. Good agreement between the MRI-derived temperature and sensor temperature was found in the tissue phantom experiment, indicating that a high-field MRI can offer good thermal precision (error < 1 °C) at a temporal resolution of 20 s per eight slices. The ability to monitor multi-slice reconstructed temperature maps offers an enhanced understanding of the temperature distribution in tissue during FUS heating.

For thermal therapies, it is required that the target temperature be reached as fast as possible and persist during the entire treatment time. Therefore, for in vivo treatment, we tested the optimal protocol first: the continuous sonication was performed for 2 min with maximum power (4.8 W/cm^2^) to bring the temperature of the tumor to 45 °C. Afterward, the power was reduced to 10% and varied in a ±3% range so that the temperature stayed at 45 °C for 30 min. All these experiments indicate the importance of the preliminary set-up of an in vivo FUS system for the safe treatment of the animals.

In most cases, determinations of the alpha value in in vivo measurements were in good agreement with the value obtained by Hindman et al. for pure water (−0.0103 ppm/°C) [28]. The fact that the estimated alpha values differ 6–8% at most from the typical value of −0.01 ppm/°C, suggests that only a 6–8% error in the MRI-derived temperature change can be explained by this potential source of error. Comparing our in vivo results with an ex vivo study by Peters et al. [29] showed similar values for untreated brain, liver, muscle, and kidney samples of both rabbit and pig. However, their setup allowed a more homogeneous heating of the samples and positioning them perpendicular to B_0_. Moreover, their results were acquired with a sagittal image orientation. Later work by Peters et al. indicated a dependence on the heat-source orientation and a geometry dependence on the PRF shift [30]. This should be considered when comparing the presented values of α to their work. One potential source of error in the temperature measurement and validation during the phantom experiments was that the heating was not homogenous over the entire target tissue. A second possible source of error in the validation was the uncertainty of the temperature probe measurements. In this study, fiber optic temperature probes were used with accuracies of 0.2 °C. 

We provided initial in vivo evidence showing the radiosensitization effect of FUS-induced hyperthermia in mouse xenografts of prostate cancer. The tumor growth of the mice which were treated with FUS + RT was suppressed with a follow-up of up to 40 days. Simultaneously, the body weight of the animals in the FUS + RT group was not significantly influenced compared to the untreated group, suggesting that the combination of FUS and RT treatment lead to no serious adverse effects on animal health. Our in vivo data showed that the combination of FUS and RT has additive effects on the treatment of prostate cancer compared to single treatments. Based on that finding, two scenarios for clinical adaptations for FUS hyperthermia treatment of the prostate gland could be realized: (i) our developed FUS transducer will be further optimized for transurethral or transrectal application to reach the prostate or (ii) existing MRgFUS prostate systems (TULSA-PRO^®^ and Exablate^®^ Prostate) designed for ablation of whole or partial gland need to be modified for HT. The additive effect of the combination therapy was also observed by Werthmöller et al. [31], where the growth of melanoma treated with hyperthermia and RT was significantly retarded compared to single RT in a mouse model. Similarly, Prasad et al. [32] reported that the combination of radiofrequency-induced hyperthermia and radiation enhances the treatment effect in mouse xenografts of human lung cancer. 

In clinical MRgHIFU treatments published previously, the real-time temperature was monitored via PRF thermometry with feedback control to the ultrasound power [2] which is also our goal for future in vivo experiments instead of using the applied offline temperature mapping. Some work groups performed FUS treatments of small animals with a dedicated set-up using a clinical MRgHIFU system (e.g., Sonalleve, Profound Medical, Canada) [8]. The MRI-compatible coil in clinical MRgHIFU systems provided high-quality images and precise temperature mapping, but the use of human scanners on a scale of small rodents is still challenging. On the one hand, most clinical scanners are still integrated into routine diagnostics, and access for research is highly limited; on the other hand, a human scanner with wide bores does not exhibit the best image quality to enable precise focusing on the target tissue, especially when it comes to orthotopic tumor models. Thus, the use of preclinical scanners and the adoption of the FUS system is preferable in comparison to the application of existing clinical systems. Additionally, in our study, a pre-clinical MRI scanner was used and PRF thermometry was visualized offline, and the real-time temperature was additionally monitored using fiber optic sensors. No obvious damages were observed in the neighboring tissues or on the mouse skin, showing that the focus spot was precisely positioned in the targeted area of the xenograft.

The inhibition of tumor proliferation and enhanced apoptosis after FUS + RT treatment compared to the other treatment groups demonstrated the sensitization effect of FUS before RT in the prostate cancer xenograft. These findings are consistent with our previous in vitro study [16]. In this context, a synergistic effect of image-guided FUS and a therapeutic agent was also reported by Wan et al. [33] in the treatment of glioblastoma in vivo. Here, the generation of reactive oxygen species (ROS) was enhanced, and thus, the anti-tumor effect was increased. The underlying mechanism for increased apoptosis levels is probably due to the generation of ROS. It is known that HT induces pro-apoptotic signaling pathways via ROS. In the case of FUS, the opening of the membrane and alteration of protein structures, especially DNA repair proteins, are also likely to contribute to the demise of the tumor cell. Similarly, Liu et al. [34] reported that a combined treatment of FUS-ablation and RT is more effective for the induction of injury in the swine pancreas compared to a single treatment. In addition, clinical evidence showed that the combination of HIFU ablation and stereotactic body radiation therapy increases the treatment efficiency in massive hepatocellular carcinomas and that the survival rates of patients were significantly enhanced compared to single treatment [35]. The future perspective of using FUS heating as an adjuvant to radiotherapy includes the idea of being able to reduce the radiation dose compared to radiation alone, e.g., only half or one-third to achieve the same effects with fewer side effects, or to have the option to re-irradiate. However, further studies are needed to determine the exact dose in combination with FUS.

## 5. Conclusions

To sum up, in our first in vivo proof-of-concept study, we successfully tested a newly developed matrix-array FUS transducer which is compatible and safe to be used in a 7 T small animal set-up. We validated the findings of preliminary in vitro data showing radiosensitization of a prostate cancer cell line PC-3 when cells are heated to 45 °C for 30 min followed by radiation within a time window of 1 h. Given that a single fraction irradiation is not common in a clinical treatment regime, further adaptions using multiple fractions, e.g., 2 Gy per week [36], and optimization towards more realistic workflows are necessary. Our in vivo results demonstrated additive effects of combined MRI-guided FUS and RT for the treatment of prostate cancer in a xenograft mouse model compared to RT alone. The combination of MRI-guided FUS and RT may provide a chance for less invasive cancer therapy through radiosensitization.

## Data Availability

All data and materials supporting the conclusion of this study have been included within the article. Original data are available on the university server.
